# Increased Body Mass Index during Therapy for Childhood Acute Lymphoblastic Leukemia: A Significant and Underestimated Complication

**DOI:** 10.1155/2015/386413

**Published:** 2015-05-25

**Authors:** Helen C. Atkinson, Julie A. Marsh, Shoshana R. Rath, Rishi S. Kotecha, Hazel Gough, Mandy Taylor, Thomas Walwyn, Nicholas G. Gottardo, Catherine H. Cole, Catherine S. Choong

**Affiliations:** ^1^School of Paediatrics and Child Health, The University of Western Australia, Perth, WA 6009, Australia; ^2^Telethon Kids Institute, The University of Western Australia, Perth, WA 6008, Australia; ^3^Department of Endocrinology, Princess Margaret Hospital, Perth, WA 6008, Australia; ^4^Department of Oncology and Haematology, Princess Margaret Hospital, Perth, WA 6008, Australia; ^5^Department of Radiation Oncology, Sir Charles Gairdner Hospital, Perth, WA 6009, Australia; ^6^PathWest, Perth, WA 6008, Australia

## Abstract

*Objective & Design*. We undertook a retrospective review of children diagnosed with acute lymphoblastic leukemia (ALL) and treated with modern COG protocols (*n* = 80) to determine longitudinal changes in body mass index (BMI) and the prevalence of obesity compared with a healthy reference population. *Results*. At diagnosis, the majority of patients (77.5%) were in the healthy weight category. During treatment, increases in BMI *z*-scores were greater for females than males; the prevalence of obesity increased from 10.3% to 44.8% (*P* < 0.004) for females but remained relatively unchanged for males (9.8% to 13.7%, *P* = 0.7). Longitudinal analysis using linear mixed-effects identified associations between BMI *z*-scores and time-dependent interactions with sex (*P* = 0.0005), disease risk (*P* < 0.0001), age (*P* = 0.0001), and BMI *z*-score (*P* < 0.0001) at diagnosis and total dose of steroid during maintenance (*P* = 0.01). Predicted mean BMI *z*-scores at the end of therapy were greater for females with standard risk ALL irrespective of age at diagnosis and for males younger than 4 years of age at diagnosis with standard risk ALL. *Conclusion*. Females treated on standard risk protocols and younger males may be at greatest risk of becoming obese during treatment for ALL. These subgroups may benefit from intervention strategies to manage BMI during treatment for ALL.

## 1. Introduction

Acute lymphoblastic leukemia (ALL) is the most common childhood malignancy. Patients within this group are at risk of energy imbalance and an increased prevalence of obesity [1]. Studies of childhood cancer survivors who were treated in the 1970s and 1980s demonstrate a clear association between cranial radiotherapy (CRT) and an increased prevalence of obesity, especially with higher radiation doses [2–4]. These effects were greatest in females and in children who were younger at diagnosis [4]. Contemporary treatment protocols have removed CRT for the majority of children with ALL and, where it is still utilized, reduced the doses delivered. Despite these improvements in treatment protocols, obesity is still a concern during treatment and follow-up [5–8]. Various risk factors have been identified including younger age at diagnosis [5, 7], elevated body mass index (BMI) at diagnosis, dose of corticosteroid [5], treatment risk [9, 10], and female sex [8, 11] but these observations are not consistent among all reports [9, 10]. In a recent meta-analysis of obesity in survivors of pediatric ALL, Zhang et al. [12] concluded that such modifiers of BMI remain speculative.

For those studies of patients on modern treatment protocols for ALL with multiple observations during treatment, few have adjusted for or included all these predictors in the same analysis [5]. Our aim was to identify those patients at greatest risk of becoming obese during treatment for ALL. We therefore performed a retrospective chart review of all children and adolescents diagnosed with ALL in Western Australia between 2003 and 2007 to evaluate longitudinal changes in BMI during treatment for ALL and to identify risk factors that would inform future intervention strategies.

## 2. Methods

### 2.1. Patient Population

**Figure 1:**
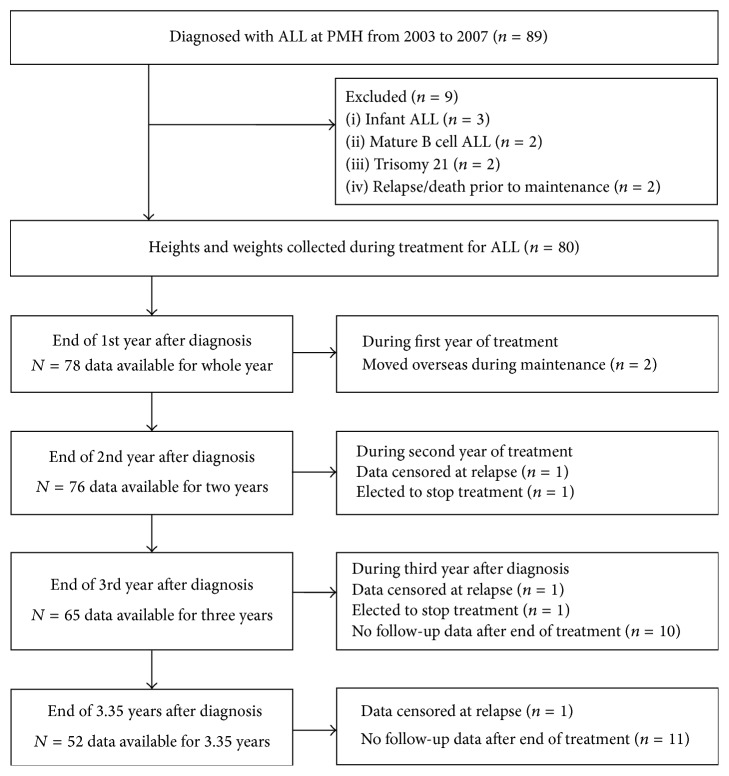
Flow diagram of patients and data included in this analysis.

Between 1 January 2003 and 31 December 2007, a total of 89 children (0–17 y) were newly diagnosed with ALL in Western Australia. All patients were treated at Princess Margaret Hospital (PMH) for Children, the sole tertiary pediatric hospital in the state of Western Australia. Patients were treated on a variety of Children's Cancer Group (CCG) or Children's Oncology Group (COG) protocols. Exclusion criteria included patients with infant ALL (*n* = 3), mature B cell ALL (*n* = 2), and trisomy 21 (*n* = 2) and patients who did not proceed to maintenance therapy due to relapse (*n* = 1) or death (*n* = 1). Thus, 80 patients were eligible for inclusion in this study (see Figure 1).

### 2.2. Data Collection from Medical Records

Medical records were retrospectively reviewed and information pertaining to auxology and cancer treatment was abstracted. Data collected included height (meters) and weight (kilograms); risk stratification, which correlates with therapeutic intensity, according to National Cancer Institute (NCI) criteria (standard/high); cumulative corticosteroid exposure; and receipt of CRT. Heights (measured using a Stadiometer) and weights were obtained at the start of each treatment phase by accredited oncology nurses for the purpose of calculating chemotherapy doses, where available heights and weights from other oncology visits were included. During treatment, height and weight were collected at least every three months. Cumulative steroid doses during treatment were calculated in mg/m^2^ for both dexamethasone (DEX) and prednisone (PDN). The total cumulative steroid dose was expressed as PDN equivalents after multiplying the DEX dose by 6.67 to account for the differences in steroid potency. For the analysis, total steroid dose was cumulated separately for the duration of maintenance treatment and for the early treatment period prior to maintenance. Exposure to cranial radiotherapy (CRT) was treated as a binary variable.

The maximum duration of treatment for any patient in this analysis was 3.35 years. Heights and weights were therefore collected for 3.35 years after the start of treatment for all patients unless data were censored at the date of relapse, transferring out of Western Australia, or transition to adult services; see Figure 1 for summary. This audit was approved by the Child and Adolescent Health Service, Quality and Safety Committee with delegated authority from the PMH Institutional Review Board. It conforms to the provisions of the Declaration of Helsinki in 1995 (as revised in Tokyo 2004) and the National Statement on Ethical Conduct in Human Research, Australian National Health and Medical Research Council.

### 2.3. The Western Australian Pregnancy Cohort (Raine) Study

Heights and weights of children from the Raine study were used as a comparison population to the ALL cohort. The Raine study (http://www.rainestudy.org.au/) has previously been described in detail [13, 14]. Briefly, 2900 pregnancies were recruited at King Edward Memorial Hospital (Perth, Western Australia) at 18 weeks' gestation from 1989 to 1991. Auxological review of this pregnancy cohort was carried out at ages 1, 2, 3, 6, 8, 10, 14, 17, and 21 years. The current Raine cohort has been shown to be representative of the ethnicity and demographics of the general population of Perth, Western Australia. This study was approved by the Raine Executive Committee and the Ethics committees of King Edward Memorial Hospital and PMH. Written consent was provided by adolescent participants and their accompanying parent or guardian. In comparison with the Raine cohort, 33% of the patient cohort were born at the same time of within 5 years of the Raine cohort, 35% of the patient cohort were born within 5–10 years of the Raine cohort, and 32% were born within 10–15 years of the Raine cohort.

### 2.4. Statistical Analyses

The outcome of interest was BMI, which was calculated using the standard formula: weight (kg)/height (m)^2^. BMI values for patients aged between 2 and 20 years were converted to age and sex-adjusted *z*-scores using the formula (((*X*/*M*)^*L*^) − 1)/*LS*, where *X* is the BMI measurement and *L*, *M*, and *S* are the age and sex specific values for the power in the Box-Cox transformation and the median and the coefficient of variation, respectively, by the SAS macros for the Center for Disease Control (CDC) 2000 growth charts [15, 16]. BMI *z*-score categories are defined as underweight (<5th percentile), healthy weight (5th percentile to <85th percentile), overweight (85th to <95th percentile), and obese (≥95th percentile). WHO 2006 growth charts were used to calculate the BMI *z*-score at diagnosis for the six children who were less than two years of age. These values were not included in the longitudinal response variable but were included in a covariate variable (BMI *z*-score at start of treatment) to ensure that all individuals were included in the statistical analysis.

Descriptive statistics were calculated using SAS 9.3. Proportions were compared using Fisher's exact test (independent samples) or McNemar's test (paired data). Longitudinal BMI *z*-scores for the ALL cohort were analyzed using linear mixed-effects, including fixed effects for time since start of treatment (quartic polynomial) and time-dependent interactions with sex, age, and BMI *z*-score at the start of treatment, NCI risk, and total maintenance steroid dose received. Interactions between sex and these covariates were explored. Random effects for intercept and time and a compound symmetry correlation structure for the errors using the* lme* function from the statistical package* nlme* in *R* were included in the models [17, 18]. *P* values were calculated for each covariate, including associated interaction terms, using the likelihood ratio test. Sample-mean predicted BMI *z*-scores were estimated using the* pred* function (package* nlme*) and associated 95% confidence intervals were calculated, which were graphed using the* ggplot2* package [19]. Longitudinal BMI *z*-scores for the Raine cohort were analyzed similarly, including fixed effects for age (quartic polynomial) and age-dependent sex interaction and random effects for intercept and age. A significance level of 5% was used in all hypothesis tests.

## 3. Results

### 3.1. Patient Characteristics

Selected characteristics of the 51 male and 29 female patients included in the study are presented in Table 1. The median age at diagnosis was 5.49 years (range: 1.02–16.66), with six children diagnosed before 2 years of age. The majority of the children were diagnosed with pre-B ALL (88.8%), of which 73% (*n* = 52/71) were standard risk. Patients were treated on CCG and COG protocols: CCG1961 (*n* = 10), CCG1991 (*n* = 24), ALL0031 (*n* = 4), ALL0232 (*n* = 12), ALL0331 (*n* = 29), and ALL0434 (*n* = 1). The duration of treatment was greater for males (median 3.18 years) than females (median 2.18 years), as males receive an additional year of maintenance therapy on these protocols. Twelve patients (15%) (10 male, 2 female) received CRT as part of their therapy. The majority of patients received DEX (*n* = 62, 78%) as the only corticosteroid, one patient received PDN only, and the remaining 17 patients received both DEX and PDN during treatment. No patient received growth hormone during treatment or in the two years immediately following the end of therapy.

### 3.2. Prevalence of Overweight and Obesity

**Figure 2:**
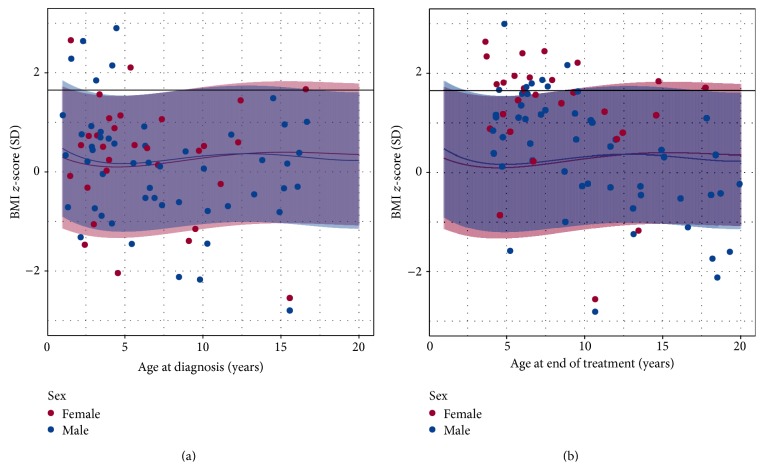
Scatter plot of BMI *z*-scores (in standard deviation (SD) units) for each individual at (a) diagnosis and (b) end of treatment. Red (female) and blue (male) shaded areas represent 95% prediction intervals from healthy (Raine study) individuals and colored lines represent mean healthy BMI *z*-scores. The black horizontal line indicates the CDC cut-off for the obese BMI category. There is a marked increase in the number of individuals in the obese category at the end of treatment.

At the time of diagnosis the BMI *z*-scores for individuals with ALL predominantly fall within the 95% prediction interval for the healthy (Raine) cohort (Figure 2(a)); 77.5% (62/80) of the patients were in the healthy weight category, 3.8% (3/80) were in the underweight category, and 8.8% (7/80) were classified as overweight and 10% (8/80) as obese according to CDC reference standards. There was no sex difference in the proportion of males and females in each of the BMI categories. At the end of treatment (Figure 2(b)), 46.3% (37/80) of the patients were in the healthy weight category, 2.5% (2/80) were in the underweight category, 26.3% (21/80) were classified as overweight, and 25% (20/80) were classified as obese. At the end of treatment, there was an increase in BMI *z*-scores for individuals with ALL diagnosed before 10 years of age, whereas individuals diagnosed after 10 years of age predominantly were within or below the 95% prediction interval for the healthy (Raine) cohort. By the end of treatment there were significant differences in the proportions of males and females in each BMI category (*P* = 0.006; Fisher's exact test). During treatment, increases in BMI *z*-scores were greater for females than males; the prevalence of obesity increased from 10.3% to 44.8% (*P* < 0.004) for females but remained relatively unchanged for males (9.8% to 13.7%, *P* = 0.7).

### 3.3. Longitudinal Analysis of BMI *z*-Scores

**Figure 3:**
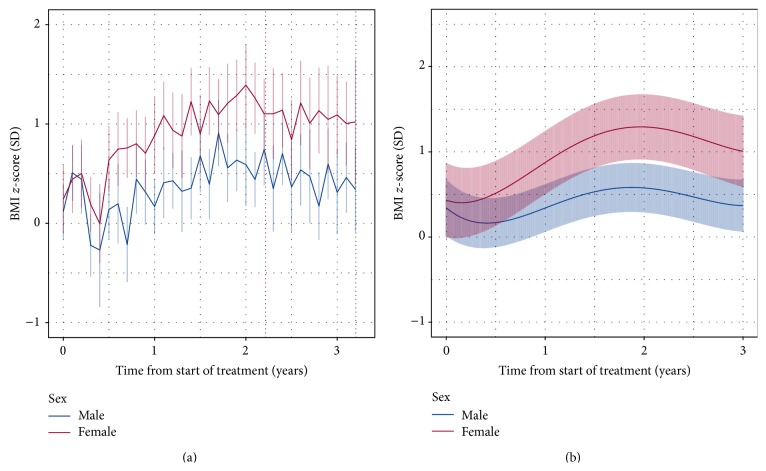
Longitudinal changes in BMI *z*-scores for males and females from the start of treatment for ALL. (a) Raw sex-specific means and 95% confidence intervals. (b) Predicted means for BMI *z*-scores and 95% confidence intervals in the mixed-effects model adjusted for sex. Vertical dotted lines indicate the mean time at the end of treatment for males and females.

The mean number of BMI observations per individual was 34.8 (SD 19.7) for females and 27.9 (SD 17.0) for males. The peak increase in BMI *z*-scores occurs around two years posttreatment, for both sexes, and thereafter gradually declines (Figure 3). The acceleration in BMI *z*-scores in females, compared to males, predominantly occurs between 6 and 12 months posttreatment, whereas from two years posttreatment an approximately constant mean difference in BMI *z*-scores is maintained between the sexes.

**Figure 4:**
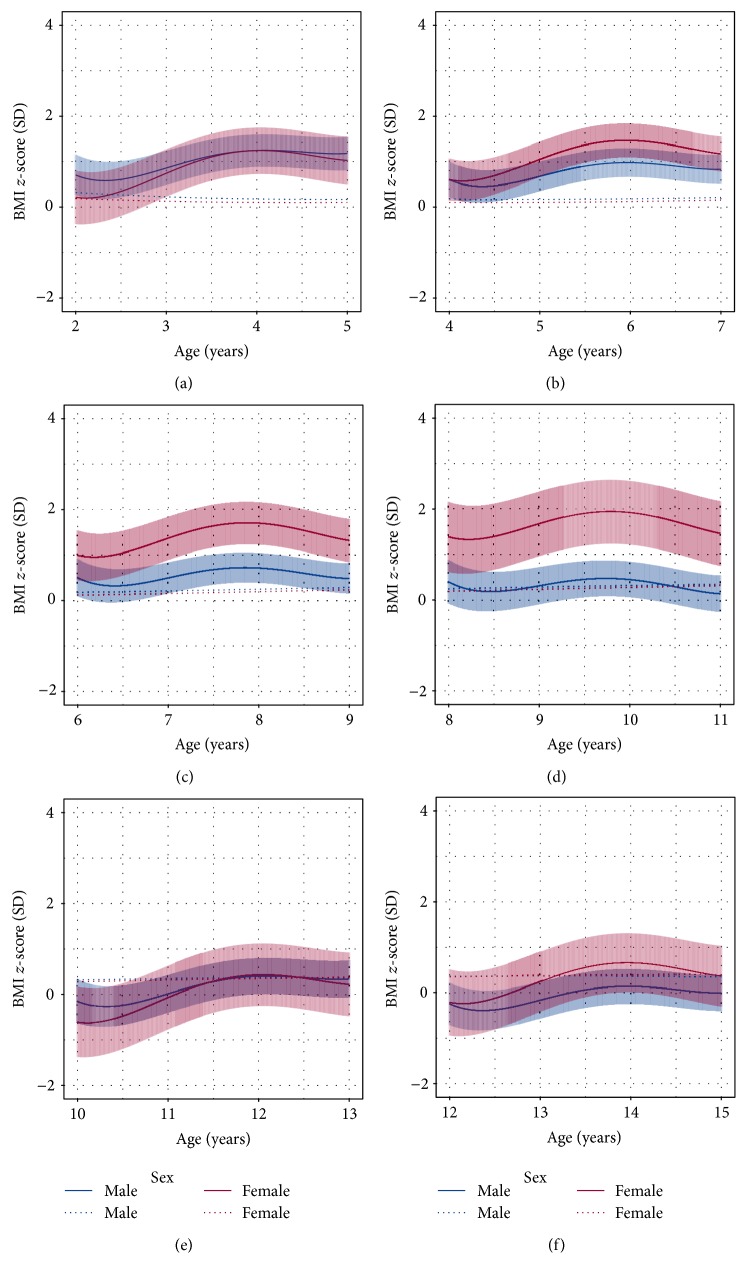
Selected scenarios in the mixed-effects model adjusted for sex, age at diagnosis, risk profile, and corticosteroid exposure. Predicted means for BMI *z*-scores and 95% confidence intervals for (a) 2 y at diagnosis with standard risk, (b) 4 y at diagnosis with standard risk, (c) 6 y at diagnosis with standard risk, (d) 8 y at diagnosis with standard risk, (e) 10 y at diagnosis with high risk, and (f) 12 y at diagnosis with high risk. Dotted lines are means of healthy (Raine study) individuals. The horizontal axes are age in years, starting from the age at diagnosis for each scenario.

BMI *z*-scores were associated with sex, NCI risk, age and BMI *z*-score at diagnosis, and total maintenance therapy steroid dose (see Table S1 in Supplementary Material available online at http://dx.doi.org/10.1155/2015/386413). No associations were detected between BMI *z*-scores and either total steroid dose in premaintenance therapy or cranial radiotherapy. Significant associations with BMI *z*-scores were identified for time-dependent interactions with sex (LRT *P* = 0.0005), BMI *z*-score at diagnosis (*P* = 0.0005), and total dose of steroid during the maintenance phase (*P* = 0.004), in addition to sex and time interactions for NCI risk (LRT *P* < 0.0001) and age at diagnosis (LRT *P* = 0.0001). These interactions are illustrated in Figure 4 and contrasted with mean BMI *z*-score for healthy (Raine) males and females at the same age. Standard risk and earlier age at diagnosis were associated with the greatest increases in mean BMI *z*-score, which is more pronounced in females. BMI *z*-scores for high-risk individuals were not significantly different from healthy individuals for either sex.  Table 2 illustrates that, on average, standard risk females have BMI *z*-scores 1 SD higher than healthy females of the same age. A similar effect size was seen in standard risk males for the youngest age group (2 years) but this effect rapidly diminished across the older age groups.

To further investigate the relationship between BMI *z*-score and steroid intake we predicted BMI trajectories based on identical steroid doses (total maintenance therapy) in males and females using the fully adjusted model described above (Figure S1 in Supplementary Material). Although slightly higher mean BMI *z*-score values are predicted with higher steroid doses, the greatest increases are based on female sex and earlier ages at diagnosis.

## 4. Discussion

Obesity is a recognized consequence of treatment for ALL; however, risk factors for this increase remain speculative [12]. Obesity may exacerbate late-effects of cancer therapy such as cardiovascular and metabolic health [1]; it is therefore important to identify survivors of ALL who are at greatest risk of becoming obese. Our longitudinal analysis which includes a median of 30 auxological measurements per patient over a period of 3.35 years has confirmed that BMI increases during treatment for ALL in childhood. The prevalence of obesity in the Western Australian ALL cohort increased from 10% at the start of treatment to 25% by the end of treatment for the whole cohort, similar to other reports of 11% to 21% [5] and 14% to 23% [8], where increases in mean BMI were expressed as *z*-scores based on the CDC reference population and increases of 0.6–1.0 SD units [6, 7, 10] are similar to our observed increase of 0.6 SD units from the start to the end of treatment. While there was a significant increase in mean BMI *z*-score during maintenance treatment in the study by Esbenshade and colleagues [9], the prevalence of obesity at the start and end of treatment (19% versus 21%) was not significantly different, which may be explained by a higher prevalence of obesity at diagnosis in this study compared with other studies.

In our cohort, female sex was a risk factor for increased BMI during treatment for ALL. In the longitudinal mixed-effects model adjusted for sex only, there were differences in predicted mean BMI *z*-scores between males and females. Although mean BMI *z*-scores increased during the first two years of treatment for both sexes, this increase was greater for females than males. Consistent with our observation, greater increases in BMI during treatment for females compared to males have been identified during treatment in several studies [5, 6, 8, 20], whereas other studies have found no differences between the sexes [9, 10, 21]. Analysis of adult BMI in one of the largest cohorts of childhood cancer survivors, the Childhood Cancer Survivor Study, has confirmed that there is a greater prevalence of obesity in adult female survivors of ALL compared to males [4]. In addition, a UK cohort treated from 1997 to 2003, which excluded patients who received CRT [6], also found increased BMI *z*-scores in females, which persisted 6 years after end of treatment [11]. Taken together, these data raise the possibility that increases in BMI during treatment for ALL in childhood persist into adulthood.

Longitudinal changes in BMI *z*-scores in children treated for ALL were associated not only with sex but also with age at diagnosis and NCI risk/therapeutic intensity. For patients with high-risk ALL, mean BMI *z*-scores did not increase with treatment irrespective of age at diagnosis or sex. For patients with standard risk ALL, there was an interaction between age at diagnosis and sex. Mean BMI *z*-scores increased for standard risk females irrespective of age but only younger standard risk males had an increase in BMI *z*-score. Thus, for standard risk patients predictors for increased BMI during treatment are younger age at diagnosis and being female. Studies typically investigate the association of BMI with either age at diagnosis or risk profile due to the potential confounding effects of age at diagnosis, which is one of the determinants of disease risk [22]. Younger age at diagnosis has been associated with increased BMI in several studies [7, 8, 23]. In addition, an association between treatment for higher risk ALL and lower BMI during treatment has also been reported [9, 10].

Corticosteroids promote increases in BMI through weight gain and disruption to height [24]. Although they are administered in high doses during treatment for ALL, few studies provide data on corticosteroid exposure during treatment for ALL, of which some [23, 25] support a role for corticosteroids in the development of obesity whereas others do not [3, 9, 26]. A recent meta-analysis [12] did not include corticosteroid exposure in its analysis due to the small number of studies with the availability of this data. In our cohort we identified a small, albeit significant, effect of cumulative steroid dose during maintenance and BMI. There was no interaction between corticosteroid dose and sex and therefore this association does not explain why females who are treated with one year less maintenance therapy compared with males have a higher prevalence of obesity by the end of treatment. Corticosteroids are widely used for other pediatric illnesses and while their use is associated with side effects such as adrenal suppression and altered bone metabolism [27–29] there are no reports of increased prevalence of obesity in females compared to males due to corticosteroid exposure.

It is known that cancer survivors who were treated in the 1970s and 1980s are at increased risk of becoming obese with risk persisting well into adulthood [4]. Children treated for ALL or brain tumors with high doses of CRT are most at risk, especially females or those who were younger at diagnosis [30, 31]. Increased risk of obesity has been associated with CRT doses ≥ 20 Gy [4] or ≥18 Gy [3]. Various studies have suggested that CRT is associated with hypothalamic damage which leads to hyperleptinemia and/or GH deficiency in young adulthood [32–35]. However, analysis of cohorts treated since 1990 that include patients exposed to CRT does not show an association with CRT and increased BMI [5, 8, 9]. Cranial radiation was not identified as a risk factor for increased BMI in our cohort. However, the 15% of the patients that received CRT were administered a relatively low (12–18 Gy) dose compared to earlier studies where over half of the study cohorts received much higher doses of CRT.

Further studies are required to elucidate the etiology of obesity in children treated for ALL on contemporary protocols, in particular why females are more susceptible to the late-effects of treatment compared with males, an effect that is consistent among a variety of cancer diagnoses and treatment protocols [31]. The CCG/COG treatment protocols for ALL typically include one year less maintenance therapy for females compared with males, yet paradoxically they are at an increased risk of obesity, suggesting that treatment regimes* per se* may not explain the sex difference and that further studies should focus on life style factors in addition to treatment modalities. Younger age at diagnosis is consistently a risk factor for development of endocrine late-effects of treatment in cancer survivors [36] including obesity. One study has reported that children diagnosed with ALL who were younger than 2.5 years of age were more likely to experience an earlier adiposity rebound which is an important risk factor in adult obesity [37]. Long-term survivors of ALL are at risk of elevated abdominal obesity [33], an atherogenic LDL phenotype [38], and diabetes [39]. These survivors have disproportionate rates of morbidity [40, 41] and mortality [36]. Additional follow-up of recent cohorts of ALL survivors beyond the end of treatment and into adulthood may determine incidence of metabolic syndrome and other morbidities. Recent studies that focus on energy balance through eating behavior and physical activity in ALL survivors suggest obesity in survivors is a result of both disordered/increased energy intake and reduced physical activity associated with fatigue, with the decrease in physical activity greater for females compared to males [42, 43].

The strengths of our study include the frequent measurement of heights and weights and analysis by longitudinal modelling. This has increased our statistical power despite the smaller size of our cohort. Thus, female patients in our cohort who received standard risk protocols for ALL as well as younger males who received standard risk protocols for ALL were at the greatest risk of becoming obese towards the end of treatment.

Our patient cohort was compared to the Raine cohort which is from the same geographical location. All children and adolescents diagnosed with ALL in the state of Western Australia are treated at PMH and therefore our cohort has 100% ascertainment of the selection criteria for the state. We have compared the changes in BMI of our ALL cohort to both the CDC reference population and the Raine cohort which is representative of the Western Australian population. Although the mean BMI *z*-scores were slightly greater in the Raine population compared to the CDC reference, the increases in the BMI *z*-scores of the cohort with ALL were greater than those observed for children in the Raine cohort over the same time frame. A third of our patient cohort was born 10–15 years after the Raine. However, a meta-analysis of trends in the prevalence of childhood overweight and obesity in Australia between 1985 and 2008 identified an increase in mean BMI *z*-score of no more than 0.1 SD units in any 3-year period with no significant differences in the rates of change between boys and girls [44]. It seems unlikely therefore that the increases in BMI observed in our patient cohort reflect secular changes in our local population.

In conclusion, this study has confirmed that there is an increased prevalence of obesity at the end of treatment for childhood ALL. In the statewide cohort of Western Australian children treated on CCG/COG protocols between 2003 and 2007, the biological risk factors for increased BMI/obesity during standard risk therapy are younger age at diagnosis and female sex. The philosophy of nutritional/dietician support in oncology units concentrates on detecting underweight children and adding high calorie feeds. While further data is required to determine the causes of obesity in survivors and the female propensity, our findings support further consideration of appropriate nutrition and energy balance during therapy and empowering of parents to exert control over excess calorie intake in their children with leukemia in the same manner as for their “healthy” children.

## Supplementary Material

Supplementary material consists of Table S1 and Figure S1.
BMI *z*-scores were associated with sex, NCI risk, age and BMI *z*-score at diagnosis, and total maintenance therapy steroid dose, multivariate regression analyses are tabulated in Supplementary Table S1. To investigate the relationship between BMI *z*-score and steroid intake we predicted BMI trajectories based on identical steroid doses (total maintenance therapy) in the mixed-effects model adjusted for sex, age at diagnosis, risk profile, this is demonstrated in selected scenarios within Figure S1.

## Figures and Tables

**Table 1:** Selected characteristics of the ALL study cohort (*N* = 80).

Age at diagnosis years	Median (min–max)
Females (*n* = 29)	4.55 (1.49–16.59)
Males (*n* = 51)	6.31 (1.02–16.66)

Risk treatment category	*N* (%)

Standard risk	53 (66%)
High risk	27 (34%)

Diagnosis	*N* (%)

Pre-B ALL	71 (88.8%)
T cell ALL	9 (11.3%)

Treatment duration years^†^	Median (min–max)

Females	2.18 (0.73–2.36)
Males	3.18 (0.75–3.35)

BMI *z*-scores	Mean ± SD (median)

Start of treatment	
Females	0.391 ± 1.080 (0.522)^#^
Males	0.163 ± 1.101 (0.186)^#^
End of treatment	
Females	1.317 ± 0.983 (1.612)
Males	0.450 ± 1.117 (0.581)

Obesity prevalence^#^	*N* (%)

Start of treatment	
Females	3/29 (10.3%)
Males	5/51 (9.8%)
End of treatment	
Females	13/29 (44.8%)
Males	7/51 (13.7%)

Obesity incidence during treatment^‡^	*N* (%)

Females	10/26 (38.5%)
Males	5/46 (10.9%)

Total dose of steroids (mg/m^2^)	Mean ± SD (median)

Premaintenance phases	
Females	2680 ± 687 (2887)
Males	2827 ± 473 (2893)
Maintenance phases	
Females	3933 ± 1293 (4087)
Males	5240 ± 2460 (6420)

Cranial radiotherapy	*N* (%)

None	68 (85%)
12 Gy	10 (12.5%)
18 Gy	2 (2.5%)

^†^2 females and 6 males who did not complete maintenance treatment due to relapse (*n* = 4), moving overseas (*n* = 2), or electing to cease treatment (*n* = 2).

^#^2 females and 4 males diagnosed before two years of age. BMI *z*-scores calculated based on CDC 2000 growth curves for age ≥ 2 years and WHO 2006 growth curves for age < 2 years.

^‡^Number of patients who became obese by the end of treatment who were not obese at diagnosis.

**Table 2:** Mean BMI *z*-scores at the end of treatment for ALL.

Age at diagnosis	Males			Females		
	ALL mean BMI *z*-score (95% CI)	Healthy mean BMI *z*-score	Difference in mean BMI *z*-score (ALL-healthy)	ALL mean BMI *z*-score (95% CI)	Healthy mean BMI *z*-score	Difference in mean BMI *z*-score (ALL-healthy)
Standard risk						
2	1.04 (0.77, 1.32)	0.17	0.88	1.23 (0.86, 1.61)	0.10	1.13
4	0.82 (0.58, 1.06)	0.21	0.61	1.25 (0.95, 1.55)	0.15	1.10
6	0.59 (0.35, 0.84)	0.29	0.31	1.27 (0.93, 1.61)	0.24	1.03
8	0.37 (0.06, 0.68)	0.35	0.02	1.29 (0.83, 1.75)	0.32	0.97
10	0.14 (−0.25, 0.54)	0.37	−0.22	1.31 (0.68, 1.93)	0.38	0.93
High risk						
10	0.30 (−0.07, 0.67)	0.37	−0.06	0.45 (−0.03, 0.92)	0.38	0.07
12	0.08 (−0.30, 0.45)	0.34	−0.26	0.47 (0.002, 0.93)	0.40	0.07

## Abbreviations

ALL: Acute lymphoblastic leukemia
BMI: Body mass index
CDC: Center for Disease Control
COG: Children's Oncology Group
CRT: Cranial radiotherapy
DEX: Dexamethasone
LRT: Likelihood ratio test
PDN: Prednisone.
