# Supplementary material for: Increased Body Mass Index during Therapy for Childhood Acute Lymphoblastic Leukemia: A Significant and Underestimated Complication
Source: Int J Pediatr. 2015 May 25;2015:386413. doi: 10.1155/2015/386413 (PMC4458559; doi:10.1155/2015/386413)
Supplement: Supplementary file 1 — Supplementary material consists of Table S1 and Figure S1. BMI z-scores were associated with sex, NCI risk, age and BMI z-score at diagnosis, and total maintenance therapy steroid dose, multivariate regression analyses are tabulated in Supplementary Table S1. To investigate the relationship between BMI z-score and steroid intake we predicted BMI trajectories based on identical steroid doses (total maintenance therapy) in the mixed-effects model adjusted for sex, age at diagnosis, risk profile, this is demonstrated in selected scenarios within Figure S1. [file 386413.f1.pdf]

Increased body mass index during therapy for childhood acute lymphoblastic leukemia: a significant and under-estimated complication

Table of contents for Supplementary Material

Table S1 Mixed effects multivariate regression analyses for BMI z-scores…………….....2

Figure S1…………………………………………………………………………………3

Selected scenarios in the mixed-effects model adjusted for sex, age at diagnosis, risk profile, if both males and females received the same maintenance steroid dose and had a BMI z-score of zero at the start of treatment. Predicted means for BMI z-scores and 95% confidence intervals for (A) 2 y at diagnosis with standard risk (B) 4 y at diagnosis with standard risk (C) 6 y at diagnosis with standard risk (D) 8 y at diagnosis with standard risk (E) 10 y at diagnosis with high risk (F) 12 y at diagnosis with high risk. Dotted lines are means of healthy (Raine Study) individuals.

Table S1: Mixed effects multivariate regression analyses for BMI z-scores

**BMI z-score (SDs)**	**Estimate**	**SE**	**p-value**
Intercept	0.548	0.282	0.05
Age (years)	-1.059	0.231	$5x10^{-6}$
Age^2 ($years^2$)	1.630	0.203	$2x10^{-15}$
Age^3 ($years^3$)	-0.751	0.0096	$8x10^{-15}$
Age^4 ($years^4$)	0.106	0.015	$1x10^{-12}$
Sex: Female	-0.735	0.274	0.009
NCI risk: High	-0.887	0.223	$2x10^{-4}$
Age at diagnosis (years)	0.021	0.023	0.36
BMI z-score at diagnosis (SD)	0.821	0.059	$<2x10^{-16}$
Total steroid maintenance dose (mg/m^2)	-0.0002	0.002	0.47
Age:Sex(Male) interaction	0.670	0.204	0.001
Age^2:Sex(Male) interaction	-0.134	0.057	0.019
Age:NCI risk interaction	0.349	0.101	$6x10^{-4}$
Sex(Male):NCI risk(high) interaction	-1.016	0.420	0.018
Age:Age at diagnosis (years) interaction	-0.045	0.011	$6x10^{-5}$
Sex(Male):Age at diagnosis (years) interaction	0.122	0.048	0.013
Age:BMI z-score at diagnosis (SD) interaction	-0.101	0.029	$5x10^{-4}$
Age:Total steroid maintenance dose (mg/m^2) interaction	0.0003	0.0001	0.004
Number of observations	2434		
Number of individuals	80		
Residual SD	0.433		
Compound symmetry Rho	-0.000000005		

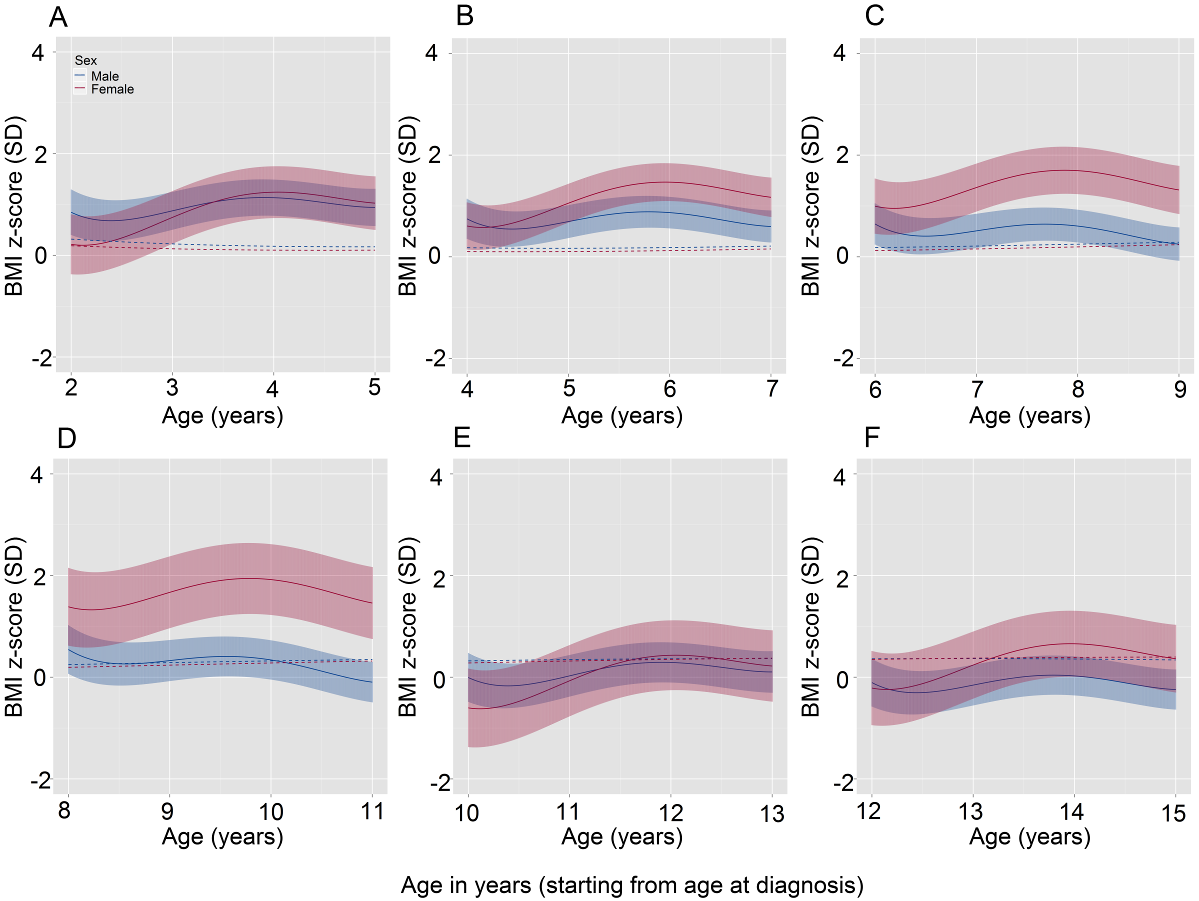

Figure S1

Selected scenarios in the mixed effects model adjusted for sex, age at diagnosis, risk profile and adjusted if both males and females received the same dose of steroids during maintenance and had a BMI z-score of zero at the start of treatment. Predicted means for BMI z-scores and 95% confidence intervals for (A) 2 y at diagnosis with standard risk (B) 4 y at diagnosis with standard risk (C) 6 y at diagnosis with standard risk (D) 8 y at diagnosis with standard risk (E) 10 y at diagnosis with high risk (F) 12 y at diagnosis with high risk ALL. Dotted lines are means of healthy (Raine Study) individuals.
